# Study of the role of Mce3R on the transcription of *mce *genes of *Mycobacterium tuberculosis*

**DOI:** 10.1186/1471-2180-8-38

**Published:** 2008-02-27

**Authors:** María P Santangelo, Federico C Blanco, María V Bianco, Laura I Klepp, Osvaldo Zabal, Angel A Cataldi, Fabiana Bigi

**Affiliations:** 1 Institute of Biotechnology, CICVyA-INTA, Los Reseros y Las Cabañas, 1712 Castelar, Argentina; 2 Institute of Virology, CICVyA-INTA, Castelar, Argentina

## Abstract

**Background:**

*mce3 *is one of the four virulence-related *mce *operons of *Mycobacterium tuberculosis*. In a previous work we showed that the overexpression of Mce3R in *Mycobacterium smegmatis *and *M. tuberculosis *abolishes the expression of *lacZ *fused to the *mce3 *promoter, indicating that Mce3R represses *mce3 *transcription.

**Results:**

We obtained a knockout mutant strain of *M. tuberculosis *H37Rv by inserting a hygromycin cassette into the *mce3R *gene. The mutation results in a significant increase in the expression of *mce3 *genes either *in vitro *or in a murine cell macrophages line as it was determined using promoter-*lacZ *fusions in *M. tuberculosis*. The abundance of *mce1*, *mce2 *and *mce4 *mRNAs was not affected by this mutation as it was demonstrated by quantitative RT-PCR. The *mce3R *promoter activity in the presence of Mce3R was significantly reduced compared with that in the absence of the regulator, during the *in vitro *culture of *M. tuberculosis*.

**Conclusion:**

Mce3R repress the transcription of *mce3 *operon and self regulates its own expression but does not affect the transcription of *mce1*, *mce2 *and *mce4 *operons of *M. tuberculosis*.

## Background

Tuberculosis (TB), a chronic illness caused by *Mycobacterium tuberculosis*, is still a major worldwide disease. Pathogenic mycobacteria species have demonstrated a remarkable ability to survive in diverse conditions encountered during the infection process. However, even after decades of investigation, there is still little knowledge about mycobacterial pathogenesis. Understanding the infective process at the molecular and cellular levels will lead to new strategies to control this disease and even to the development of an effective vaccine.

The analysis of the complete sequence of the *M. tuberculosis *H37Rv genome revealed the presence of four paralogous *mce *genes, all encoded in an operon structure consisting of eight genes [1]. The biological function of Mce proteins is not known, but increasing evidence has demonstrated that they are clearly related to the virulence of *Mycobacterium tuberculosis *complex species [2-8].

Gene regulation is considered to play a central role in host-microbe interactions, and many virulence genes are regulated in response to the host. Casali and collaborators [9] identified a regulatory mechanism which controls *mce1 *expression. They have demonstrated that a homologue of the FadR subfamily of GntR transcriptional regulators, *Rv0165c *(designated Mce1R), is a negative regulator that intracellularly represses expression of the *mce1 *operon. In addition, a gene encoding a putative transcriptional factor, *Rv0586*, is located immediately upstream of *mce2 *operon and it is transcribed in the same direction as that of *mce2 *genes. Furthermore, it has been found that there are growth phase and tissue specific differences in the expression of *mce *operons in *M. tuberculosis *[10-12] which is in agreement with the presence of regulatory mechanisms controlling *mce *transcription. In a previous work, we found evidence indicating that *Mce3R*, a TetR family transcriptional regulator, down-regulates the *mce3 *operon during the *in vitro *growing of *M. tuberculosis *[13]. We have demonstrated that the overexpression of Mce3R in both *M. smegmatis *and *M. tuberculosis *abolishes the expression of a gene reporter fused to *mce3 *promoter.

TetR family members often regulate their own synthesis [14-18]. The classic example of self regulation in members of this family protein is a repressor involved in resistance to tetracycline of *Escherichia coli*, which has given the name TetR to the group [19]. In a number of TetR-autoregulated systems the regulator and the structural genes are divergently transcribed and the region for protein binding overlaps the promoters placed in the intergenic region [15,18,20]. That is the case of *mce3R*, which is placed upstream of *mce3 *operon, oriented in the opposite direction and separated from it by a region of 880 bp.

In this work we validate the role of Mce3R in repressing the *mce3 *transcription in *M. tuberculosis *by analyzing gene expression in a *mce3R*-knockout *M. tuberculosis *strain. We also found that this regulation is exclusive for the *mce3 *operon among *mce *genes and that the Mce3R repressor regulates its own expression.

## Results

### Construction of a *mce3R *mutant in *M. tuberculosis*

**Figure 1:**
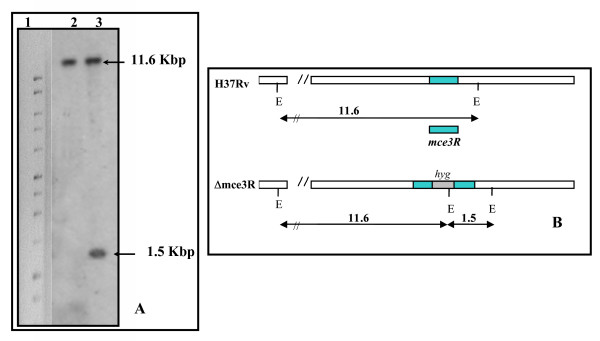
**Disruption of the *mce3R *gene of *M. tuberculosis *H37Rv**. (A) Southern blot analysis of chromosomal DNA from sucR counterselected tuberculosis clone (lane 3) and parental strain (lane 2). Genomic DNA was digested with EcoRI and hybridized to the *mce*3R probe. Arrows indicate position of hybridizing fragments. MWM 1 kb Promega is shown on the left (lane 1). (B) Restriction map of mutant and wild type strains. The insertion of hygromycin-resistant cassettes is indicated (*hyg*). Arrows represent the length of expected bands after digestion with EcoRI (E). Value on each arrow indicates the molecular weight of expected bands expressed in base pairs (bp).

As a first step to assess the *mce3 *operon expression in the absence of Mce3R, we obtained a knockout mutant strain of *M. tuberculosis *H37Rv by inserting a hygromycin cassette into the *mce3R *gene. The site-directed mutant strain of *M. tuberculosis *was obtained by two-step mutagenesis strategy by using the p2NIL shuttle plasmid [21], which carries the *lacZ *gene and the counter selectable marker *sacB*. Allelic exchange was confirmed in the selected clones (Hy^R^, Km^S^, and Sac^R^) by Southern blotting (Fig. 1A), since the mutant showed a hybridizing fragment of about 1.5 kb absent in the wild-type strain. This polymorphism is due to the introduction of an extra EcoRI site present in the hygromycin cassette (Fig. 1B). The mutant strain was designated Δ*mce3R*. The mutation was complemented by transforming the plasmid pSummce3R into the mutant.

### *In vitro *characterization of Δmce3R

**Figure 2:**
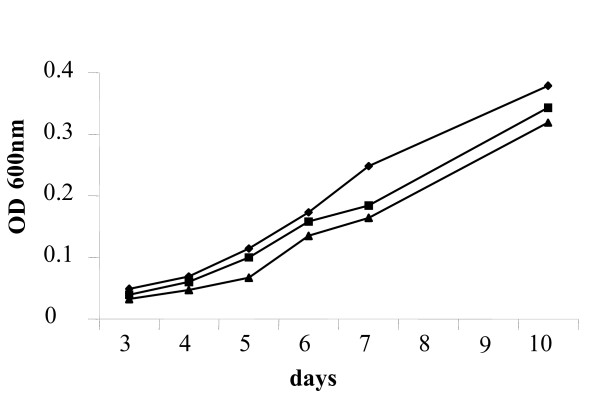
**Effect of the *mce3R *mutation on *in vitro *growth of *M. tuberculosis***. Cultures of the mutant [Δmce3R, square], the complemented [Δmce3R (pSummce3R), triangle] and the parental wild-type [H37Rv, rhombus] strains were grown to stationary phase and inoculated into fresh Dubos medium supplemented with 0.4% glucose at OD_600nm _0.005 and the OD_600nm _was measured at various time points. It is shown a representative experiment from triplicate.

To determine whether *mce3R *disruption introduces alterations during *in vitro *growth, growth curves of the Δ*mce3R *mutant, complemented, and parental strains were compared under standard culture conditions. All assayed strains showed similar doubling time and growth characteristics throughout the culture period (Fig. 2). This result indicates that the mutation does not affect the *in vitro *growth of *M. tuberculosis*.

### *mce3 *operon expression is repressed by Mce3R during *in vitro *culture and inside murine macrophages

We have previously demonstrated that the *mce3 *promoter allows the expression of the *lacZ *reporter gene in *M. tuberculosis *H37Rv but that this expression is completely abolished when Mce3R is overexpressed in the H37Rv strain from a multi-copy plasmid [13]. Although these findings constitute initial evidence demonstrating the role of Mce3R as a repressor of the *mce3 *operon transcription, the presence of an endogenous copy of the *mce3R *gene in *M. tuberculosis *did not enable us to determine the conditions in which the regulator system operates.

**Figure 3:**
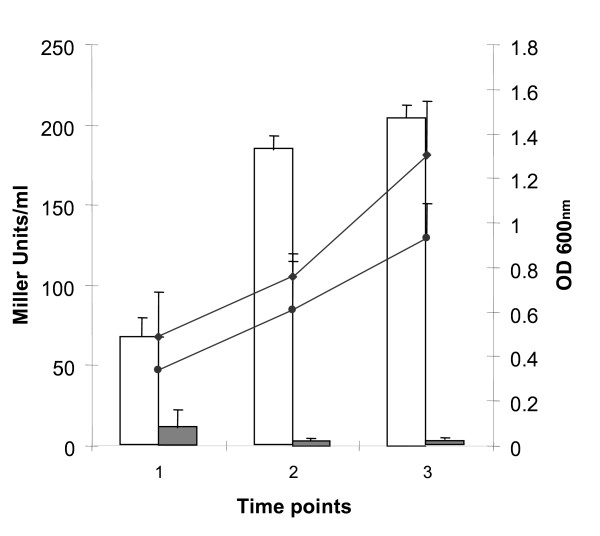
**Effect of Mce3R on *mce3 *promoter activity during the growth of *M. tuberculosis***. Comparison of *mce3 *promoter activity during the growth of Δmce3R (pP3-mce3R-lacZ) (grey bars) and Δmce3R (pP3-lacZ) (white bars) strains in M7H9-AD-G medium. The results are presented as β-galactosidase activity expressed as Miller units ± S.D. of duplicate in three time points (1 early exponential phase, 2 exponential phase and 3 stationary phase). Growth curves of Δmce3R (pP3-lacZ) (square) and Δmce3R (pP3-mce3RlacZ) (circle) strains are shown and the OD 600_nm _values are indicated on the right. Results represent one of at least three independent experiments.

Here, in order to compare the expression the *mce3 *operon either in the absence or in the presence of Mce3R, DNA fusions of the *mce3 *promoter to *lacZ *reporter, containing or not containing *mce3R *were cloned within pYUB178-*lacZ*. The resulting plasmids, pP3-mce3R and pP3 respectively, were integrated into the chromosome of the Δmce3R strain. The β-galactosidase activity was measured at different points along cultures of *M. tuberculosis *grown *in vitro *and in a macrophages cell line. Since transcription of *mce3 *genes has previously shown to be increased when *M. tuberculosis *was grown in rich media [10-12] the expression of *mce3 *operon was assessed in both synthetic (7H9) and rich (Dubos) media (Figure 3 and data not shown).

While hardly any β-galactosidase activity was detected either in *in vitro *cultures (Fig. 3) or in cell-line mice macrophages (Table 1) in the presence of Mce3R (Δmce3R::pP3-mce3R strain), the activity in the Δmce3R::pP3 strain was remarkably high in all of the conditions tested. The promoter activity increased along the *in vitro *cultures of Δmce3R:: pP3, and peaked at 24 h of infection inside the cell. These results clearly indicate that Mce3R represses the expression of *mce3 *operon in *M. tuberculosis *in the growth conditions tested.

**Table 1:** Effect of MceR on *mce3 *promoter activity in *M. tuberculosis *during infection of J774 Macrophage-like cell line.

	***β*-galactosidase activity^a^in J774 **cell line		
**Strain**	**4 h**	**24 h**	**72 h**
**Δmce3R (pP3-*mce3*R)**	0	0	0
**Δmce3R (pP3)**	114+/-5	2571+/-172	2162+/-92

^a ^calculated as: arbitrary β-galactosidase units/number of bacteria (CFU)/100000.

Values are presented as the mean and standard deviation (SD) of reactions performed in triplicate.

### Assessment of the role of Mce3R in the transcription of the four *mce *operons

A number of reports indicate that the expression of all *mce *genes depends on the growth conditions [10-12]. These observations, together with the findings that regulatory proteins are involved in the expression of the *mce1 *and *mce3 *operons, suggested the idea of a broader regulatory mechanism differentially controlling the expression of *mce *genes. In order to test whether Mce3R is able to control the transcription of the other *mce *operons apart from *mce3*, the expression of one gene from each *mce *operon in the mutant strain was compared with that of the wild type. Primers were designed to amplify a 189, 168, 234, 151 and 134 bp region on *mce1D *(*Rv0172*)*, mce2A *(*Rv0589*), *mce3E *(*Rv1970*), *mce4A *(*Rv3499*), and *sigA *respectively. Amplicons of expected size were obtained with each pair of primers (data not shown).

**Figure 4:**
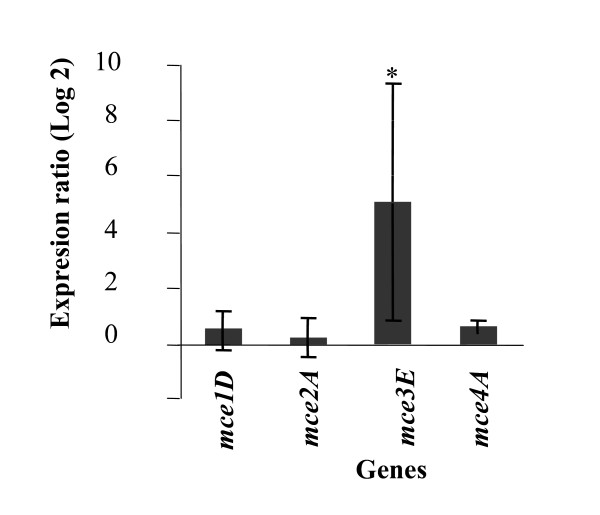
**Effect of Mce3R on transcription of *mce *operons during *in vitro *growth of *M. tuberculosis***. The data are presented as the fold change in gene expression in mutant Δmce3R strain normalized to *sigA *endogenous reference gene and relative to the wild type H37Rv strain +/- SD of derived results from four independent experiments. *Significant differences (P < 0.05) of gene expression in both strains as calculated by Pair Wise Fixed Reallocation Randomisation.

Differences in relative gene expression between the wild type and the mutant strains were assessed in group means for statistical significance by a randomisation test (see Materials and Methods). While the relative abundance of *mce3E *mRNA was significantly higher (P < 0.030) in the mutant than in the wild type strain in the conditions evaluated, no significant differences between both strains were observed on the expression of *mce1D *(P < 0.74), *mce2A *(P < 0.918) and *mce4A *(P < 0.511) (Fig 4).

To verify that RNA samples were not contaminated with genomic DNA, RT-PCR reactions were performed without the addition of reverse transcriptase. The lack of amplification products verifies that the RT-PCR products were amplified from RNA that had been reversely transcribed into cDNA.

These results demonstrate that Mce3R regulates exclusively the transcription of *mce3 *operon among *mce *genes in the conditions tested

### Mce3R expression is self regulated

**Figure 5:**
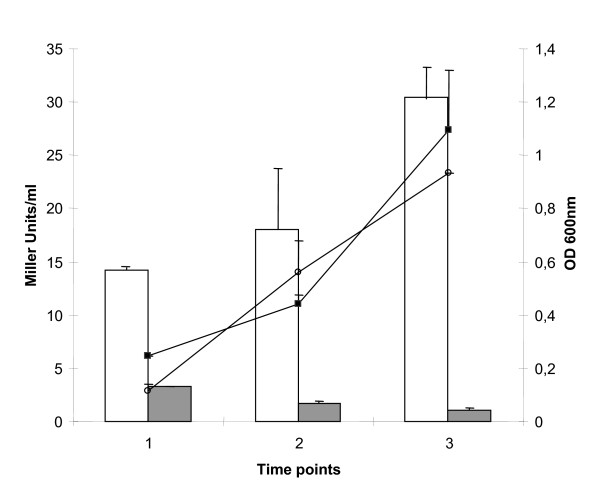
**Effect of Mce3R on its own transcription during *in vitro *growth of *M.tuberculosis***. Comparison of *mce3R *promoter activity during the growth of Δmce3R (pPR3-lacZ) (white bars) and H37Rv (pPR3-lacZ- Mce3R endogenous) (grey bars) strains in M7H9-AD-G medium. The results are presented as β-galactosidase activity expressed as Miller units ± S.D. of duplicate in three time points (1 early exponential phase, 2 exponential phase and 3 stationary phase). Growth curves of Δmce3R (pPR3-lacZ) (circle) and H37Rv (pPR3-lacZ- Mce3R endogenous) (rhombus) strains are shown and the OD 600_nm _values are indicated on the right. Results represent one of three independent experiments.

To investigate whether *mce3R *is subject to transcriptional autoregulation, a transcriptional fusion was constructed between the *mce3R *promoter and the *lacZ *reporter. Since *mce3R *is located adjacent to *mce3 *operon and divergently oriented, the *mce3R *promoter is situated in *mce3R*-*yrbE3A *intergenic region. The entire intergenic region was fused to the *lacZ *gene within pYUB178-*lacZ *to create plasmid pPR3-*lacZ*. The pPR3-*lacZ *plasmid was transformed into the wild type *M. tuberculosis *H37Rv and the *M. tuberculosis *Δmce3R mutant, and β-galactosidase activity was measured to assess the levels of *mce3R *promoter activity with and without Mce3R regulator. As shown in figure 5, in the presence of Mce3R (wild type H37Rv strain) the activity of *mce3R *promoter is steadily and significantly reduced as compared with that in the absence of the Mce3R regulator (mutant Δmce3R strain). This reduction in *mce3R *promoter activity was more evident during the stationary growth phase. These experiments demonstrate that the Mce3R protein is able to transcriptionally repress expression of the *mce3R *promoter in *M. tuberculosis *during the *in vitro *culture condition tested.

## Discussion

Little is known about gene regulation of virulence factors in *M. tuberculosis *due to its slow growth rate and the late development of mycobacterial genetics. Prokaryotic transcriptional regulators are classified in families on the basis of sequence similarity and structural and functional criteria. The TetR family, a family of transcriptional regulators that is well represented and widely distributed among bacteria, has a helix turn-helix (HTH) signature, the most recurrent DNA binding motif, to bind its target DNA. Members of the TetR family of repressors control transcription of proteins involved in multidrug resistance, enzymes implicated in different catabolic pathways, biosynthesis of antibiotics, osmotic stress, and pathogenicity of gram-negative and gram-positive bacteria. At least 40 putative TetR-family regulator genes are spread on the *M. tuberculosis *genome. Most of them are similar to the TetR/AcrR family, but just a few have been characterized. Mce3R was the first TetR-like regulator studied in *M. tuberculosis *[13]. Then, Engohang-Ndong *et al*. [22] found that a member of the TetR/CamR family represses the expression of *ethA *that encodes a protein that catalyses the activation of ethionamide (ETH). ETH is an important second-line anti TB drug used for the treatment of patients infected with multidrug-resistant strains.

The transcription profile of the *mce3 *operon in different *in vitro *growth conditions of *M. tuberculosis *has been addressed by RT-PCR in a number of publications and transcription of *mce3 *genes has been found when bacteria were cultured both on LJ and Dubos media [11,12] but not in 7H9 synthetic medium in both exponential and stationary growth phases [10]. Using similar methodology we detected mRNA of *mce3 *operon during *in vitro *culture of *M. tuberculosis *either in synthetic or in rich (data not shown) media. However, transcription from the *mce3 *promoter, measured as β-galacosidase activity, was completely absent in the presence of Mce3R both in *in vitro *conditions and inside a murine macrophages cell line. These last results, together with the finding that the elimination of Mce3R significantly increases *mce3 *transcription, indicate that the potential level of *mce3 *expression is repressed in the conditions of growth assayed.

Interestingly, the high homology among *mce *operons is not conserved among their regulator genes, since Mce1R, the other *mce *regulator described, as well as the putative regulator of the *mce2 *operon, belong to the GntR family [9,1]. In addition, no putative regulatory gene is placed in the vicinity of the *mce4 *operon; however, it was recently proposed Rv3574, a TetR-type regulator, as represor of *mce4 *operon expression [23].

Here it was demonstrated that *mce3R *is not involved in the expression of *mce1*, *mce2 *nor *mce4 *operons. Therefore, it is tempting to speculate that both facts, i.e. gene redundancy and differential regulation, ensure the production of Mce proteins in different environments.

Here we demonstrated that *mce3 *promoter is stronger than *mce3R *promoter, and *mce*3 expression seems to be mostly shut down during *in vitro *growth, but it is also likely that under unknown particular conditions of growth it would turn on.

As it happens to many other proteins of the TetR family, we found that Mce3R negatively regulates its own expression. The experiments with the *mce3R *promoter indicate that there is in average ten folds decreased in transcriptional activity when the Mce3R is provided. In this condition, the activity of *mce3 *promoter is sixty times repressed. Since the *mce3R *– *yrbE3A *intergenic region spans 880 bp, it is likely that Mce3R binds to consensus motifs located between the divergent genes in both promoter regions.

Although this study provides more insights to the role of Mce3R in the regulation of the *mce *operons, the information is still limited and further studies are necessary to detect any other gene regulated by this system. Elucidation of other promoters targeted by Mce3R will lead to the definition of a consensus *mce3R*-binding site and the possibility of define the Mce3R-regulon. Thus, one could hope to decipher the function of *mce3 *genes by the identification of the Mce3R regulon.

## Conclusion

The available evidence demonstrates that while Mce3R represses powerfully the transcription of *mce3 *operon *in vitro *and inside macrophages, it does not affect the transcription of *mce1*, *mce2 *and *mce4 *operons during *in vitro *culture of *M. tuberculosis*. It was also demonstrated that Mce3R negatively regulates its own expression but the level of expression is lower than that observed for *mce3 *operon.

## Methods

### Bacterial strains and culture media

All cloning steps were performed in *Escherichia coli *DH5α. Regulation studies were performed in *M. tuberculosis *H37Rv. *E. coli *was grown in Luria-Bertani (LB) broth or on LB agar. *M. tuberculosis *strains were grown in Middlebrook 7H9 medium supplemented with 0.05% Tween 80, Dubos and Middlebrook 7H11, all supplemented with albumin 0.5%, dextrose 0.4%), and 0.5% glycerol (M7H9-AD-G). When necessary, 20 μg kanamycin ml^-1 ^and 50 μg hygromycin ml^-1 ^were added to the media.

### General DNA methodology

PCR amplifications from genomic DNA templates were performed as previously described [13]. Each primer contained base mismatches that introduced a restriction site suitable for directional cloning (Table 2). Chromosomal DNA samples were obtained as described vanSoolingen [24]. Purification of plasmids and DNA fragments were performed using the GFX Micro Plasmid Prep Kit (GE Healthcare) and DNA and Gel Band Purification Kit (GE Healthcare), respectively, according to the manufacturer's instructions. Plasmid pYUB178-*lacZ *was created by insertion of the β-galactosidase gene from plasmid pMC1871 (AmershamPharmacia) into *Hind*III and *Nhe*I sites of pYUB178 mycobacterial integrative vector [25]. *M. tuberculosis *H37Rv and *M. tuberculosis *Δmce3R (see below) were transformed by electroporation, as described by Parish and Stoker [26].

**Table 2:** Primer sequences used in this study.

**Primer**	Sequence*
P3rev	ggatccggcgcggcgcaccagctggattcga
*mce*3R-P3up	ggatccggacacctcattcacaccgataatg
upMutReg	gcggccgcgagcgggaggtgaccaaggc
lowMutReg	gcggccgcgaagaaggccgacgcgaagc
upP3mce3Rint	tcatgaggacacctcattcacaccgataat
P3mce3Rrevint	tcatgaaccatggcgcggcgcaccagctggat
*upP3int*	aagcttttgcgcaccggaatcacaaatc
*P3revint*	aagcttaccatggcgcggcgcaccagctggat
*mce*3Eup	gacaccttcaccgcatacct
*mce*3Elow	ggtggtcttgttgaccgagt
*sig*A1	ggccagccgcgcacccttgac
*sig*A2	gtccaggtagtcgcgcaggacc
*mce*1Dup	ggcaagggtaagcaaatcaa
*mce*1Dlow	ggtcaacctgtcggtgaact
*mce*2Aup	gaagaccgagctgactatgg
*mce*2Alow	atgtagcgaggattcacgtc
*mce*4Aup	ggtaggcaaggtcacggata
*mce*4Alow	aatgaattccaccgatttgg
P3anti up	aagcttggcgcggcgcaccagctgga
P3anti low	aagcttaccatttgcgcaccggaatcaca

* Restriction enzyme site added at the end of each primer is underlined.

### Construction of *M. tuberculosis *Δmce3R mutant strain

A genomic region containing *mce3R *and about 2 kb flanking 5' and 3' regions was obtained by PCR from *M. tuberculosis *H37Rv total DNA by using primers: upMutReg and lowMutReg. The amplified fragment was cloned in site *Not*I of p2NIL plasmid [21] and the mutant allele of *mce3R *was generated by inserting a cassette conferring hygromycin resistance from pUC-Hy7 (AmershamPharmacia) into a unique *Hind*III site internal to *mce3R*. The final delivery vector was generated by incorporation of the *Pac*I cassette from pGOAL 17 into this last p2NIL recombinant vector. Mutants were constructed using a two-step strategy as described previously [21]. Chromosomal DNA was prepared from the selected clones and digested with *Eco*RI and then analyzed by Southern blotting by using the wild-type gene as probe. The mutant strain resulting from allelic exchange was designated *M. tuberculosis *Δ*mce3R*.

DNA fragment encompassing *mce3R *and the intergenic region between *mce3R *and *Rv1964 *was PCR amplified with primers: mce3R-P3up and P3rev and cloned into TOPO 2.1 vector (Invitrogen). A fragment containing *mce3R *and its promoter was released from this last plasmid by digestion with *Eco*RI and *Bam*HI and cloned into pSUM41 [27] to produce plasmid pSummce3R. This plasmid was used to transform *M. tuberculosis *Δmce3R strain by electroporation. The resulting complemented strain was referred to as Δ*mce3R*::*mce3R*.

### RNA preparation

Culture pellets of 50 ml were resuspended in 1 ml of TRIzol (Invitrogen). Cells were disrupted using a Fastprep FP120 bead-beater (Savant) for 20 s at a speed of 6.0 m s^-1 ^with Lysing Matrix B (Q-Biogene). Then, 200 μl of chloroform (Merck) was added, and the mixture was incubated for 15 min at room temperature. Tubes were centrifuged at 10,000 × *g *for 15 min at 4°C, and the supernatant was extracted again with 100 μl of chloroform and alcohol precipitated with 600 μl of isopropanol and 60 μl of 3 M ammonium acetate (pH 5.3) at -70°C overnight. Pellets were washed with 75% ethanol and resuspended in 50 μl of diethyl pyrocarbonate-treated water (Sigma-Aldrich). The RNA preparations were treated with DNase amplified grade (Invitrogen).

### RT-Q-PCR

DNA-free RNA (1 μg) extracted from middle logarithmic-phase culture of either *M. tuberculosis *H37Rv or Δmce3R was mixed with 50 ng of random primers (Invitrogen) in 20 μl of final volume and reversely transcribed to total cDNA with SuperScript III reverse Transcriptase (Invitrogen) following the manufacturer's instructions. Identical reactions lacking reverse transcriptase were also performed to confirm the absence of genomic DNA in all samples.

Q-PCR was performed in the Applied Biosystems 7000 DNA sequence detection system (Perkin-Elmer Corp.), by using Master Mix QuantiTect SYBR Green (Qiagen), 1 μl of template cDNA and the pairs of primers listed on Table 1. Each reaction was performed in duplicate. Results were presented as ratios calculated with the Relative expression software tool (REST@) application described by Plaffl et al. [28]. Relative quantification of each target (*mce*) gene was performed by using *sigA *as reference gene and a subsequent test for significance of derived results was performed by using Pair Wise Fixed Reallocation Randomisation [29]. The value of PCR efficiency for all transcripts was 2, as calculated following the formula: E = 10^[-1/*slope*]^.

### Construction of β-galactosidase fusions

DNA fragments encompassing the intergenic region between *mce3R *and operon *mce3*, either containing or not containing the coding sequence of *mce3R*, were generated by PCR amplification with the pairs of primers upP3mce3Rint/P3mce3Rrevint and upP3int/P3revint, respectively. Both DNA fragments were cloned into the *Hind*III or the *Nco*I sites of p178-lacZ, giving rise to plasmids pP3-lacZ and pP3-mce3R-lacZ, respectively. The intergenic region was also amplified by PCR using primers P3antiup/P3antilow and the DNA fragment was cloned in plasmid pYUB178-*lacZ *to generate pPR3-*lacZ*. This last plasmid is the antisense version of pP3-*lacZ *in which the promoter of *mce3R *was fused to the reporter gene. These plasmids were used to transform *M. tuberculosis *strains as indicated.

### Measurements of β-galactosidase activity

Determination of β-galactosidase activity in *M. tuberculosis *recombinant strains was performed as previously described [13]. Briefly, β-galactosidase activity was measured in soluble cell extract prepared from aliquots of *in vitro *cultures taken at different time points. Results were expressed in Miller units [*A*420 × 1000/reaction time (min) xA600] [30].

Cultures of the murine macrophage-like cell line J774 were infected with recombinant *M. tuberculosis *H37Rv strains (free of clumps) at a m.o.i. of 5. J774-infected cells were disrupted with 1% Triton ×-100, at 4, 24 and 72 h post-infection. β-galactosidase measurements were performed on the soluble cell extract by using the Chemiluminescent lacZ β-galactosidase detection kit (MGT Product M08550) and the Luminometer, Veritas 1.4 (Turner Biosystems, Inc.). β-galactosidase activity was related to the number of bacteria as determined by bacterial counting.

## Authors' contributions

MPS constructed the *M. tuberculosis *mutant strain and the plasmids, FCB performed the RT-QPCRs, MVB and OZ carried out cell infection experiments, AC participated in the design of the study and performed the statistical analysis, LK and FB conceived of the study, and participated in its design and coordination and drafted the manuscript. All authors read and approved of the final manuscript.
